# Phenolic Profiles and Biological Activities of Extracts from Edible Wild Fruits *Ehretia tinifolia* and *Sideroxylon lanuginosum*

**DOI:** 10.3390/foods10112710

**Published:** 2021-11-05

**Authors:** Imelda N. Monroy-García, Irma Edith Carranza-Torres, Pilar Carranza-Rosales, María Oyón-Ardoiz, Ignacio García-Estévez, Jesús Fernando Ayala-Zavala, Javier Morán-Martínez, Ezequiel Viveros-Valdez

**Affiliations:** 1 Deparatmento de Química, Facultad de Ciencias Biológicas, Universidad Autónoma de Nuevo León, Av. Pedro de Alba S/N, San Nicolás de los Garza 66450, Nuevo León, Mexico; Imelda.monroygrc@uanl.edu.mx (I.N.M.-G.); irma.carranza@uanl.edu.mx (I.E.C.-T.); 2 Centro de Investigación Biomédica del Noreste, Instituto Mexicano del Seguro Social, Jesús Dionisio González #501, Col. Independencia, Monterrey 64720, Nuevo León, Mexico; carranza60@yahoo.com.mx; 3 Grupo de Investigación en Polifenoles, Departamento de Química Analítica, Nutrición y Bromatología, Facultad de Farmacia, Campus Miguel de Unamuno, Universidad de Salamanca, E37007 Salamanca, Spain; m.oyon@usal.es (M.O.-A.); igarest@usal.es (I.G.-E.); 4 Coordinación de Tecnología de Alimentos de Origen Vegetal, Centro de Investigación en Alimentación y Desarrollo A.C., Carretera Gustavo Enrique Astiazarán Rosas No. 46, Hermosillo 83304, Sonora, Mexico; jayala@ciad.mx; 5 Facultad de Medicina, Universidad Autónoma de Coahuila Unidad Torreón, Gregorio A. García No. 198, Torreón 27000, Coahuila, Mexico; javiermoranmartinez@uadec.edu.mx

**Keywords:** wild fruits, antioxidant, phenolic compounds, HPLC-DAD-MS/MS

## Abstract

*Ehretia tinifolia* Linnaeus (Boraginacea) and *Sideroxylon lanuginosum* Michaux (Sapotaceae) are wild fruits consumed in North America and are appreciated for their pleasant flavor and sweet taste. However, details regarding their composition and biological properties in the available literature are scarce. This study reports the phenolic composition, antioxidant, antiproliferative activities, and digestive enzymatic inhibition of amberlite-retained methanolic extracts from both fruits. Results revealed that these wild fruit extracts are rich in antioxidants. *S. lanuginosum* had lower phenolic but higher flavonoid contents (21.4 ± 1.5 mg GAE/100 g FW and 6.42 ± 0.9 mg CE/100 g FW) than *E. tinifolia* (64.7 ± 2.6 mg GAE/100 g FW and 5.1 ± 0.4 mg CE/100 g FW). HPLC-DAD-MS/MS analysis showed rosmarinic acid as a major polyphenol in *E. tinifolia* and quercetin glucoside in *S. lanuginosum*. Polyphenols content in *E. tinifolia* was related to a significant free radical scavenging ability: DPPH (EC_50_ = 0.32 ± 0.03 mg/mL), TEAC (4134 ± 9.7 μM TE/g dry extract), and hemolysis inhibition (IC_50_ = 58.55 ± 2.4 μg/mL). Both extracts were capable of inhibiting *α*-glucosidase, partially inhibiting *α*-amylase, and showed no inhibition against lipase, while showing antiproliferative activity against HeLa, HT-29 and MCF-7 cancer cell lines. Our study revealed that these wild fruit extracts are rich in health-beneficial phytochemicals and hold significant potential for elaborating functional foods.

## 1. Introduction

In recent years, exploratory studies have been developed to identify endemic wild fruits as promising crops with high economic value [1]. Likewise, producing scientific evidence of the properties of these wild resources is highly relevant to evaluating their contribution to local biodiversity and assessing their effects on nutrition and human health [2,3]. *Ehretia tinifolia* Linnaeus (Boraginacea), commonly called pinguica and *Sideroxylon lanuginosum* Michaux (Sapotaceae), known as Gum Bully, Black Haw, or Coma, are wild fruits appreciated for their pleasant flavor and sweet taste and have been consumed in North America by Native Americans since pre-Columbian times. Thus, wild plants have an important role in indigenous peoples’ lives [4] as they supplement staple foods to provide a balanced diet to many populations in several regions across Mexico, mainly in the states of Michoacán, Nayarit, Nuevo León, San Luis Potosí, Sinaloa, Tamaulipas, and Veracruz. It has also been used in the southeastern USA, mostly used by the Kiowa and Comanche tribes [5]. *E. tinifolia* are small globoid yellow drupes of up to 8 mm diameter, with a sweet flavor and have been used as food and medicinal plant in several regions of Mexico and the USA [6,7,8,9]. Bromatological analysis of *E. tinifolia* has already been reported, and the content of total phenols in polar organic extracts (50 to 125 mg per 100 g of fresh fruit) is related to its antioxidant properties [10]. *S. lanuginosum* is also used in the north of Mexico and by native tribes of the USA as chewing gum [11]. However, there are no reports of the chemical composition or biological activity of this fruit. Therefore, expanding the chemical/biological knowledge of these species would add value to the possible production and commercialization of nutraceuticals or functional foods.

It is well known that fruits have antioxidants with a beneficial impact on human health. Phenolic compounds, ascorbate and carotenoids are the main antioxidants found in fruits [12]. The main phenolic components present in fruits are glycosylated flavones/flavonols, flavanones, anthocyanins, and phenolic acids [13]. Epidemiological studies and randomized clinical trials showed a strong association between polyphenol consumption and reduced risk of several chronic diseases, including cancer, diabetes, and inflammatory processes [14]. Dietary plant polyphenols modulated metabolism of carbohydrates and lipids, controlled hyperglycemia, dyslipidemia and insulin resistance, increased metabolism in adipose tissues, and alleviated oxidative stress and stress-sensitive signaling pathways and inflammatory processes in several in vitro, animal models and some human studies [12]. Furthermore, cancer prevention is one of the most documented biological properties of polyphenols. In cancer, these compounds induced apoptosis, reduced the number of tumors, inhibited angiogenesis, modulated multidrug resistance and antiproliferative activity [15]. Polyphenols also modulated the expression of cytochrome P-450 enzymes and activation of carcinogens [16].

Wild fruits with antioxidant activity have also become the object of an increasing number patent claims on behalf of the food and beverage industry. Wild rose fruit and jujube mixtures have been used to claim the formulation of an antioxidant beverage [17]. In addition, wild pawpaw has been used to formulate cakes [18]. At the same time, wild indigo fruit and wild blueberries have been used to formulate beers claiming to enhance vision and human immunity [19]. Wild rose fruit rich in polyphenols has been used to formulate cosmetics with skin repair activity [20]. Similarly, wild cherries were used to formulate beverages claiming to be useful on dietary therapies [21]. Additionally, Berbenol^®^ (PharmExtrcta, Pontenure, Italy), a tablet formulation made from an extract of *Berberis aristata* D.C. and *Silybum marianum* (L.) Gaertn fruit is used to treat glycemia and lipid value alterations in patients with type 2 diabetes [22]. These patents evidence the potential economic impact of knowing the bioactive composition of wild fruits.

In this context, this study aimed to identify the phenolic profiles and contents from *E. tinifolia* and *S. Lanoginosum* from the north of Mexico and to evaluate the antiradical, antidiabetic and anticarcinogenic properties. This information would highlight their potential use in elaborating functional foods and preserving and revalorizing their use as traditional foods.

## 2. Materials and Methods

### 2.1. Plant Material

Ripe and healthy pinguicula and coma fruits were collected by hand from wild trees located in San Nicolas de los Garza, N.L. (northeast Mexico; Latitude: 25°44′31.9″ N; Longitude: 100°16′59.3″ W) and Higueras, N.L. (northeast Mexico; Latitude: 25°57′59.3″ N; Longitude: 100°00′52.5″ W), respectively, from May to August 2019. Fruits were immediately transported to the laboratory for processing. The authenticity of the collected species was confirmed by Dr. Marco Guzmán Lucio, taxonomist and responsible for the herbarium of the Faculty of Biological Sciences of the Autonomous University of Nuevo Leon.

### 2.2. Phytochemicals Extraction

The method used for phytochemicals extraction was reported previously by Viveros-Valdez, et al. [23]. *E. tinifolia* and *S. lanuginosum* fresh fruits were washed with distilled water, and seeds were removed manually. One hundred grams of each fruit pulp and peel were squeezed and mixed with 1 L of distilled water using a commercial blender. The mixtures were filtered using Whatman No.1 filter paper, and each juice was applied onto an Amberlite XAD-7 column (150 × 10 cm), which had been conditioned previously with 2 L of distilled water. The XAD-7 column was washed with distilled water (3 L) to eliminate proteins, carbohydrates, minerals and organic acids, while the retained phenolic compounds were eluted with methanol (MeOH) (2 L). Subsequently, the solvent was evaporated until dryness under reduced pressure and lyophilized for analysis. Yellow and purple extracts were obtained from *E. tinifolia* (*w/w* yield: 0.87%) and *S. lanuginosum* (*w/w* yield: 0.51%), respectively. Lyophilized extracts were stored at 4 ºC for a maximum period of 6 months.

### 2.3. Determination of Total Phenolic Content (TPC)

Total phenolic content was determined using the Folin-Ciocalteu reagent as described by Singleton and Rossi [24] with some modifications. Total phenolic content includes free and bound phenolics. The freeze-dried extracts were dissolved in distilled water to a concentration of 0.1 mg/mL. Then, 30 μL of each fruit extract, 150 μL of Folin-Ciocalteu reagent (1:10 *v/v* in distilled water) and 120 μL of an aqueous solution of Na_2_CO_3_ (7.5% *w/v*) were placed into a 96-well plate and incubated for 30 min at room temperature in darkness. Absorbance was measured at 765 nm (Agilent BioTek Epoch Microplate Spectrophotometer), using gallic acid solutions as standards (concentrations range of 10–1000 µg/mL in distilled water) (y = 0.0681x + 0.0732; R^2^ = 0.9848), and results were expressed as milligrams of gallic acid equivalents (GAE) per 100 g of fresh weight (FW). Data were reported as mean ± S.D. for at least three replications.

### 2.4. Determination of Total Flavonoid Content (TFC)

Total flavonoid content was determined using the aluminum chloride colorimetric assay [25]. Fruit extracts were diluted with MeOH (1 mg/mL). Then, 250 μL of fruit extract or standard solution of (+) Catechin (concentration range of 50–500 µg/mL of MeOH) was mixed with 1000 μL of distilled water and 75 μL of NaNO_2_ solution (7% *w/v* in distilled water). After 5 min at room temperature, 75 μL of AlCl_3_ aqueous solution (10% *w/v*) were added. One minute later, 500 μL of 1 M NaOH and 600 µL of distilled water were added and vigorously mixed. The absorbance in the reaction mixture was measured at 496 nm (Agilent BioTek Epoch Microplate Spectrophotometer). The values of the calibration curve are y = 0.0124x + 0.0173; R^2^ = 0.9995. Results were expressed as mg (+) Catechin Eq./100 g of fresh weight (FW). Data are reported as mean ± S.D. for at least three replications.

### 2.5. Antiradical and Antioxidant Assays

#### 2.5.1. 2,2′-Diphenyl-1-Picrylhydrazyl Radical (DPPH^●^) Assay

The ability of the phytochemical extracts to scavenge the 2,2′-diphenyl-1-picrylhydrazyl radical (DPPH^●^) was assessed following the methodology proposed by Braca, et al. [26] with some modifications. Fruit extract (1 mg/mL of MeOH) was added to a 150 µM methanol solution of DPPH^●^ in a serial dilution 1:1 (*v/v*) ratio. Absorbance at 517 nm was determined after 30 min in darkness using a microplate spectrophotometer (Agilent BioTek Epoch) and converted into the percentage of antiradical activity (AA) using the following formula:

AA% = 100 − [(As − Ac) × 100/Ac]

where Ac and As are the absorbance of the control and samples, respectively. MeOH was used as a negative control and Trolox as the positive control. Mean values were obtained from triplicate experiments. The radical scavenging activities were expressed as the median effective concentration (EC_50_) (mg/mL). The EC_50_ was calculated from the log-dose inhibition curve obtained by a nonlinear regression algorithm.

#### 2.5.2. Trolox Equivalent Antioxidant Capacity (TEAC) Assay

The procedure followed the method of Gupta et al. [27] with slight modifications. An aqueous solution of the radical was prepared with 7 mM ABTS and 2.45 mM potassium persulfate dissolved in distilled water and kept for 16 h at room temperature in darkness. The solution was then diluted by mixing ABTS^●+^ solution with deionized water to obtain an absorbance of 0.7 ± 0.02 units at 734 nm using a microplate reader (Agilent BioTek Epoch). In a 96-well microplate, 20 µL of fruit extracts were allowed to react with 200 µL of the ABTS^●+^ solution for 20 min in darkness. Then the absorbance was measured at 734 nm. The standard curve was linear between 25 and 600 mM Trolox (y = 0.1095x − 9.8189; R^2^ = 0.9521). Results are expressed in μmol of Trolox equivalents (TE)/g of dry extract.

#### 2.5.3. Protective Effect on Human Erythrocytes

Hemolysis was induced by peroxyl radicals generated by AAPH (2-2′-Azobis (2-amidinopropane dihydrochloride) according to the methodology of Silva-Beltrán et al. [28]. Then, 5 mL of blood was obtained from healthy human volunteers by venipuncture and collected into tubes containing EDTA as an anticoagulant. Erythrocytes were separated from plasma by centrifugation at 1500 rpm for 10 min at room temperature and washed three times with five volumes of phosphate-buffered saline (PBS) (37 mM of NaCl, 2.7 mM of KCI, 8 mM of Na_2_HPO_4_, and 2 mM of KH_2_PO_4_ *w/v*) at pH 7.4. Later, erythrocytes were suspended in four volumes of PBS solution to obtain a density of 8 × 10^9^ cells/mL.

The addition of AAPH to the suspension of washed erythrocytes induces oxidation of membrane lipids and proteins, resulting in hemolysis. The erythrocyte suspension (250 µL) was mixed with 250 µL of fruit extracts dissolved in PBS at concentrations of 500, 750, 1000, 2000 µg/mL and 250 µL of 300 mM AAPH in PBS. The reaction mixture was shaken gently while being incubated at 37 °C for 3 h. After the incubation, the reaction mixture was diluted with eight volumes of PBS and centrifuged at 4000 rpm for 5 min. The supernatant’s absorbance was measured in a microplate reader (Agilent BioTek Epoch, Santa Clara, CA, United States) at 540 nm. Percent inhibition was calculated by the following equation:

% Inhibition = [A_AAPH_ − As]/[A_AAPH_ × 100]

where A_AAPH_ is the absorbance of AAPH at 540 nm and As is the absorbance of the extracts at 540 nm. The extract concentration providing 50% inhibition (IC_50_) was also calculated from the dose-response curve obtained by plotting the percentage of hemolysis inhibition versus the extract concentration. Ascorbic acid was used as positive control and PBS as a negative control. Three independent experiments were used for these calculations.

### 2.6. Digestive Enzymes Inhibition

#### 2.6.1. Inhibition of α-Glucosidase

The α-glucosidase inhibitory activity was evaluated according to the chromogenic method described by Kaskoos [29], with some modifications. Then, 50 µL of serial dilutions of 5 mg/mL of fruit extract dissolved in PBS solution (pH 6.8, DMSO 1%, *v/v*) was mixed with 50 µL of α-glucosidase (0.8 U/mL of PBS, pH 6.8). Then, the 96-well plate was incubated at 37 °C for 15 min. After that, 50 µL of 625 mM *p*-nitrophenyl-α-D-glucopyranoside (PNPG) solution was added to each well and incubated for another 15 min. Subsequently, 100 µL of 0.2 M Na_2_CO_3_ were added to stop the reaction and absorbance was measured at 405 nm using a UV-visible microplate reader (Agilent BioTek Epoch). The percent inhibition was calculated using the following formula:

% Activity = [Ac − As/Ac] × 100

where Ac and As are the absorbance of control and sample, respectively, and acarbose was used as positive control and PBS solution as a negative control. The half-maximal inhibitory concentration (IC_50_) was calculated using a logit analysis.

#### 2.6.2. Inhibition of α-Amylase

The α-amylase inhibitory activity was evaluated according to Sudha et al. [30] with some modifications. α-Amylase (1 U/mL) dissolved in PBS (pH 6.8) was mixed with serial dilutions (with 5 mg/mL) of fruit extract dissolved in PBS solution (pH 6.8, DMSO 1%, *v/v*) in a 1:1 dilution (*v/v*) and incubated in a 96-well plate at 37 °C for 15 min. Then, 50 µL of 0.5% starch solution in PBS was added to each well, and the reaction was incubated for 20 min at 37 °C. The reaction was stopped with 20 µL of 1 M HCl, followed by the addition of 50 µL of iodine reagent (3 mM I_2_ and 30 mM KI), and absorbance was measured at 750 nm using a UV-visible microplate reader (Agilent BioTek Epoch). The percent inhibition was calculated using the following formula:

% Activity = [Ac − As/Ac] × 100

where Ac and As are the absorbance of control and sample, respectively, and acarbose was used as a positive control and PBS solution as a negative control. The IC_50_ values were calculated using a logit analysis.

#### 2.6.3. Inhibition of Pancreatic Lipase

The pancreatic lipase inhibitory activity was determined according to Maqsood et al. [31] with slight modifications, using *p*-nitrophenyl palmitate (*p*-NPP) as a substrate. Under reaction conditions, the lipase enzyme hydrolyses *p*-NPP to release *p*-nitrophenol, a yellow-colored substance and can be measured at 410 nm. Pancreatic lipase (2.5 mg/mL) was dissolved in phosphate buffer solution PBS (60mM, pH 8). In a 96-well microplate, 100 μL of serial dilutions of fruit extract (5 mg/mL) or Orlistat was mixed with 30 μL of lipase solution. It was incubated for 15 min at 37 °C. Then, 10 μL substrate *p*-NPP (10 mM in DMSO) was added. After incubating the mixture for 30 min at 37 °C, its absorbance was measured at 405 nm in a microplate reader. The percent inhibition was calculated using the following formula:

% Activity = [Ac − As/Ac] × 100

where Ac and As are the absorbance of control and sample, respectively. The control contained all constituents except a test sample. Orlistat was used as a positive control. The IC_50_ values were calculated by logarithmic regression analysis.

### 2.7. Antiproliferative Activity

The antiproliferative effects of extracts of *E. tinifolia* and *S. lanuginosum* were assessed using the method described previously by Viveros-Valdez, et al. [32]. HeLa (ATCC**^®^** CCL-2), MCF-7 (ATCC**^®^** HTB-22) and HT-29 (ATCC**^®^** HTB-38) cancer cell lines were used. The cells were seeded in 25 cm^2^ tissue culture flasks in Dulbecco’s modified eagle medium (DMEM) mixed with Ham’s nutrients (Ham’s F-12) and supplemented with 10% fetal bovine serum (FBS), adjusting the pH to 7.2. A mixture of antibiotics composed of penicillin and streptomycin (10,000 IU/mL: 10,000 µg/mL; 1 mL of mixture/1 L of medium) was added. Cells were maintained at 37 °C in an incubator under a 5% CO_2_/95% air atmosphere at constant humidity. Cells were harvested by trypsinization, counted with a hemocytometer, and their viability was confirmed by Trypan Blue (0.4%). The different cancer cell lines were seeded with 5000 cells per well in a 96 well plate. After incubation for 24 h, 100 µL of fruit extracts (concentrations of 125, 500, 750, 1000, and 2000 µg/mL dissolved on PBS (pH 7.2) were added and incubated for another 24 h. 20 µL of Alamar Blue Invitrogen™ (Waltham, MA, USA) solution (10% *v/v*) was added to each well. The plate was incubated with agitation and the fluorescence was measured 4 h later in a fluorometer FLx800 Bio-Tek Instruments, INC (Waltham, MA, USA) (535 nm excitation and emission at 595 nm wavelength). Taxol was used as a positive control. The IC_50_ values were calculated by probit analysis.

### 2.8. HPLC–DAD-ESI-MS/MS Analysis of Phenolics Profiles

The phenolic composition was performed using a Hewlett-Packard 1100 Series liquid chromatograph (Agilent Technologies, Waldbronn, Germany) equipped with a Spherisorb S3 ODS-2 (80 Å, 3 mm, 4.6 mm × 150 mm) C-18 reversed-phase column (Waters Corporation, Milford, MA, USA) thermostatted at 35 °C. Optimization of the HPLC conditions was carried out for the analysis of these samples. The mobile phases employed were 0.1% (*v/v*) formic acid in water (solvent A) and 100% HPLC-grade acetonitrile (solvent B). The elution was performed at a flow rate of 0.5 mL/min, and the gradient was established as follows: from 0% to 10% B for 3 min, from 10% to 14.5% B for 34 min, from 14.5% to 20% B for 3 min, from 20% to 35% B for 15 min and from 35% to 60% B for 5 min. The absorption spectra were recorded between 220 and 600 nm, and detection was conducted at 250, 280, 330, and 370 nm as the preferred wavelengths. The HPLC system was coupled via the DAD outlet with a mass spectrometer API 3200 (AB Sciex LLC, Framingham, MA, USA) equipped with an electrospray ionization source and a triple quadrupole linear ion trap mass analyzer, controlled by the Analyst 5.1 software. Mass spectrometry detection was performed in negative mode as previously described by Cittadini et al. [33] for the analysis of flavanols and phenolic acids: declustering potential, 40 V, entrance potential, and ion spray voltage were set at 40 V, 7 V, and 5000 V, respectively whereas GS1 GS2 and curtain gas were set at 40 psi, 50 psi, and 20 psi, respectively, with collision gas as “high”. A full mass analysis (collision energy 10 V) and an MS^2^ analysis (collision energy 25 V) were performed. The phenolic characterization was analyzed in 1 mg of dry extract of each fruit resuspended in deionized water. Retention time, UV-vis spectra, parent ion, and fragmentation pattern data were used for compound identification. The differences in the relative abundance of the fragment ions obtained in MS/MS analysis of each compound were compared to the fragmentation patterns reported in the literature to differentiate between isomers.

### 2.9. Statistical Analysis

The values were expressed as mean ± SD (*n* = 3). The observed differences among means were performed using one-way analysis of variance (ANOVA), followed by Tukey’s pairwise comparison of means. The statistical analysis was carried out by one way analysis of variance using SPSS (version 18) statistical analysis program. Statistical significance was considered at *p* < 0.05.

## 3. Results and Discussion

### 3.1. Total Phenolic Content (TPC), Total Flavonoid Content (TFC), Antiradical, and Antioxidant Activity

The TPC was higher for *E. tinifolia* (64.7.4 ± 2.6 mg GAE/100 g F.W.) compared with *S. lanuginosum* (21.4 ± 1.5 mg GAE/100 g F.W.) (*p* < 0.05) (Table 1). *E. tinifolia* extract showed higher TPC than ethanolic extracts of wild fruits *Prunus spinosa*, *Rosa canina* and *Rubus sanctus* (0.29 ± 0.02, 0.23 ± 0.02 and 0.42 ± 0.02 mg GAE/g F.W., respectively) [34]. Otherwise, Pio-León et al. [10] evaluated the phenolics content of *E. tinifolia* fruits, and they found higher concentrations in the methanolic extract (125.45 ± 2.9 mg/100g F.W.) than in the ethanolic extract (50.25 ± 6.85 mg/100g F.W.). Schmeda-Hirschmann et al. [35] determined the TPC for *Sideroxylon obtusifolium,* and it was lower (4.71 ± 0.06 g GAE/kg F.W.) than *S. lanuginosum* in this study. The TPC on *E. tinifolia* is comparable with wild fruits as *Eulychnia acida Phil*. (80.6 ± 2.2 mg GAE/100 g F.W.) [36], *Garcinia atrovidiris* (68.45 ± 0.9 mg GAE/100g F.W.) and *Durio zibenthinus* (64.57 ± 3.43 mg GAE/100g F.W.) [37].

TFC was higher for *S. lanuginosum* (9.85 ± 1.6 mg CE/100 g F.W.) than *E. tinifolia* (6.4 ± 0.2 mg CE/100 g F.W.) (*p* < 0.05) (Table 1). Both fruit extracts showed higher TFC than *Litchi chinensis* (6 ± 1 mg CE/100g F.W.) and *Citrus reticulata* (4 ± 1 mg CE/100 g F.W.) and *Persea americana* (2 ± 1 mg CE/100g F.W.) [38]. However, the flavonoid content of different solvents extractions of *Ceratonia siliqua* fruits fluctuated between 0 to 98.7 ± 2.4 mg CE/100 g F.W. These results suggest that the total flavonoid content was strongly affected by the extraction [39]. Therefore, the low flavonoid contents obtained may be due to the extraction method.

The present work evaluated the antiradical and antioxidant activity of the samples using three different assays, including in vitro DPPH^●^ and TEAC assays and ex vivo inhibition of hemolysis in erythrocytes induced by AAPH. The antioxidant activity is summarized in Table 1. In all determinations, the most active extract was *E. tinifolia*. The antioxidant activity correlated with total phenolic compounds on this extract compared to *S. lanuginosum*. The DPPH^●^ antiradical activities of *E. tinifolia* (EC_50_ = 0.32 ± 0.03 mg/mL) and *S. lanuginosum* (EC_50_ = 0.48 ± 0.05 mg/mL) were higher than reported for other wild fruits. Pulp and peel extracts from *Cydonia oblonga* fruit showed DPPH^●^ free radical scavenging activities EC_50_ of 0.6 and 0.8 mg/mL, respectively [40] and XAD7 extract from *Prumnopitys andina* reported an EC_50_ of 0.93 ± 0.03 mg/mL [41]. For the TEAC assay, *E. tinifolia* showed a lower TEAC value (2454 μM/g) than *S. lanuginosum* (4134 μM/g) (*p* < 0.05). Both fruits presented high antiradical activity regarding other wild fruits such as *Prunus espinosa* (5080 μM/g), *Rubus ulmifolius* (4810 μM/g) and *Arbutus unedo* (4480 μM/g) [42].

The measurement of oxidative hemolysis in erythrocyte membranes represents a good model to study antioxidant and pro-oxidant compounds. AAPH was used as a peroxyl-radical generator to induce hemolysis in human erythrocytes. The protective capacity of *E. tinifolia* and *S. lanuginosum* extracts on human red blood cells, using AAPH radical showed values of IC_50_ of 58.55 μg/mL and 61.76 μg/mL, respectively (Table 1). Our results showed similar IC_50_ values compared to epicatechin (IC_50_ value of 42.3 μg/mL) [43], a known antioxidant flavonoid that has reported beneficial health effects [44,45]. Compared to other fruit extracts, such as *Cydonia oblonga Miller* (IC_50_ = 652 μg/mL) [40] and *Mangifera indica L*. (520 μg/mL shown 35% of hemolysis inhibition) [46] the extracts from *E. tinifolia* and *S. lanuginosum* presented higher antioxidant capacity.

### 3.2. HPLC-DAD-MS/MS Analysis of Phenolic Profiles

**Figure 1:**
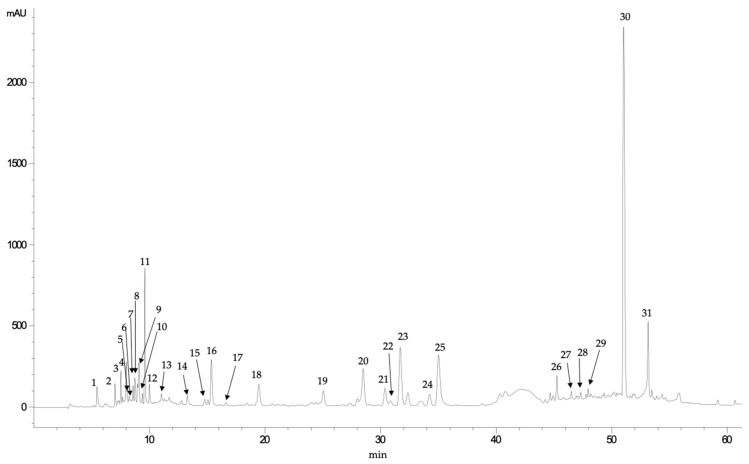
Chromatogram (registered at 280 nm) obtained in the analysis of phenolic compounds of pinguica (*E. tinifolia*).

*E. tinifolia* fruits (Figure 1) contained mainly derivatives of both hydroxybenzoic acids, such as gallic and syringic acids, and hydroxycinnamic acids, such as caffeic acid. However, it was observed that the most abundant compounds in *E. tinifolia* extract produced, in their mass analysis, *pseudomolecular* ions at *m/z* 359 and 537, which can be attributed to rosmarinic acid (RA) and different lithospermic acid derivatives, respectively (Table 2). Thus, compound 30, which showed a retention time of 51.02 min (Figure 1), was identified as RA, based on its parent ion (m/z 359) and its fragmentation pattern, which was as follows: fragment at *m/z* 359 corresponded to the RA radical ion [M-H-C_18_H_16_O_8_]^−^; fragment at *m/z* 197, to the radical ion of 3,4-dihydroxyphenylactic acid [M-H-C_9_H_10_O_5_]^−^; and fragment at *m/z* 179, to the radical ion of caffeic acid [M-H-C_9_H_8_O_4_]^−^. Other fragment ions obtained for this compound were fragment at *m/z* 161 ([M-H-C_9_H_7_O_3_]^−^), which can be attributed to acylonium ion and fragment at *m/z* 135, corresponding to [M-H-C_8_H_6_O_2_]^−^. These same fragments were found in compounds 27, 28, and 29, except they showed other signals that corresponded to fragments not determined as sugars. Compounds 20 to 25 were identified as lithospermic acid derivatives, which were isomers since they showed the same *pseudomolecular* ion at *m/z* 625, with the main fragment ion detected at *m/z* 537 (C_27_H_22_O_12_), which can be identified as lithospermic acid. Moreover, the fragment ion at *m/z* 493 [M-H]^−^ can be formed by the loss of CO_2_ (44 Da) of the ion at *m/z* 537, whereas fragment ions at *m/z* 295 [M-H-CO_2_-C_9_H_10_O_5_]^−^ and *m/z* 312 [M-H-C_9_H_8_O_4_]^−^ were derived from the ion at *m/z* 493 due to the loss of C_9_H_10_O_5_ and C_9_H_8_O_4_ fragment ions_._ These fragments are those reported by Huang et al. [47] for lithospermic acid. Both RA and lithospermic acid derivatives could be relevant for the biological activity of these extracts since RA has been reported to be a compound with significant antioxidant and antineoplastic activity [32], and the lithospermic acid and its mono- and dimethyl esters are known to inhibit adenylate cyclase [48].

**Figure 2:**
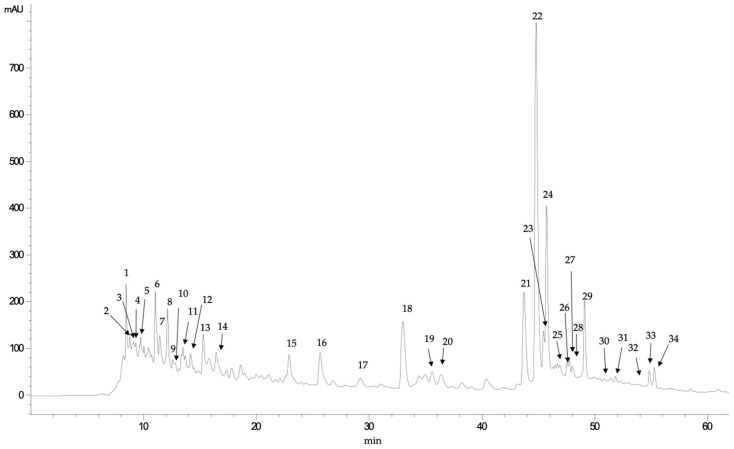
Chromatogram (registered at 280 nm) obtained in the analysis of phenolic compounds of coma (*S. lanoginosum*).

In the case of *S. lanoginosum* fruits (Figure 2), although different hydroxybenzoic acids (such us gallic and protocatechuic acids) and hydroxycinnamic acids (such as coumaric and ferulic acids) were identified, the main family of phenolic compounds identified was flavonols. Thus, mainly quercetin and myricetin glycosides and their corresponding non-glycoside forms were identified in these fruits (Table 3). The structure of flavonoids often results in substituents such as hydroxyl, methyl, and methoxyl groups. Therefore, in the MS/MS analysis, fragment ions are usually derived from the loss of CO (28 Da), H_2_O (18 Da) or CO_2_ (44 Da) molecules, as well as the fragment ions of substituents [49]. In addition, the retro-Diels–Alder (RDA) fragmentation is a common fragmentation pattern in flavonoids. Compound 34, which showed a *pseudomolecular* ion [M-H]^−^ at *m/z* 301, was identified as quercetin (C_15_H_10_O_7_). The fragment ion at *m/z* 273 [M-H-CO]^−^ was derived from the loss of a CO (28 Da) and that at *m/z* 179 ([M-H-C_7_H_6_O_2_]^−^) can be explained by the RDA fragmentation. Subsequently, fragment ions at *m/z* 151 [M-H-C_7_H_6_O_2_-CO]^−^ originated from the ion at *m/z* 179 by the loss of a CO (28 Da) [46]. Compound 22 was the main compound, and it was identified as quercetin glucoside, showing a *pseudomolecular* ion at *m/z* 463, whose fragmentation produced the ion at *m/z* 301 [M -H C_15_H_10_O_7_]^−^ after the loss of a glucoside molecule (162 Da). Similarly, compounds 23 and 24 also showed fragment ions at *m/z* 301 in their MS/MS analysis, formed after losing a glucuronic acid (176 Da). In contrast, compounds 25 and 26 were identified as quercetin-pentoside because of detecting the same ion fragment (*m/z* 301) after the loss of 132 Da (which can be attributed to a pentose moiety). Quercetin derivatives and, mainly quercetin glucoside, can be relevant for the biological activity of *S. lanoginosum* fruits, since this compound has been shown to protect DNA and erythrocytes from oxidative damage and exhibited anticancer activity [50].

### 3.3. Digestive Enzymes Inhibition

#### 3.3.1. Inhibition of α-Glucosidase

Currently, only a few α-glucosidase inhibitors, such as acarbose and voglibose, have been approved to treat diabetes, and their structures are mainly composed of sugar moieties [51]. Thus, many studies have been focused on searching for alternative α-glucosidase inhibitors with non-sugar core structure, particularly the polyphenols, due to their abundant availability in nature and their promising biological activities [52]. In the α-glucosidase assay, *E. tinifolia* and *S. lanuginosum* extracts showed high inhibitory activities with IC_50_ values of 0.17 and 0.21 mg/mL, respectively (Table 4). Under the same experimental conditions, the positive control acarbose presented an IC_50_ value of 0.13 mg/mL. Lopez-Martinez et al. [53] evaluated onions extracts for the inhibition of α-glucosidase activity and found that white variety inhibited more than 50% at 0.7 mg/mL. Hogan et al. [54] reported that red grape extract (1.5 mg/mL) inhibited 47% of α-glucosidase enzyme activity, and the inhibition potency was significantly higher than the white grape extract they tested (1.5 mg/mL), which only inhibited α-glucosidase activity by 39%. This research shows that an extract with more phenolics and anthocyanins is a better α-glucosidase inhibitor. Furthermore, it has been demonstrated that quercetin and kaempferol in *S. lanuginosum* and rosmarinic acid present on *E. tinifolia* are effective inhibitors of this enzyme [55,56]. Considering these compounds as the main polyphenols of extracts activity, the mechanism of α-glucosidase inhibition by wild fruit extracts could be due to the non-competitive or mixed-type interactions that polyphenols such as quercetin, kaempferol [57,58,59], and rosmarinic acid [60] maintain with the enzyme. Further studies must be done to determine the mechanism of action of *S. lanuginosum* and *E. tinifolia* extracts. These evidences emphasize the possible application of wild fruits or their derivatives in the design of diabetes treatments.

#### 3.3.2. Inhibition of α-Amylase

α-Amylase enzyme is one of the key enzymes in the human digestive system, which degrades starch to monosaccharides and causes the rise of blood glucose [61]. Natural amylase inhibitors offer an attractive therapeutic approach to the treatment of postprandial hyperglycemia by decreasing glucose released from starch. Extracts of *E. tinofolia* and *S. lanuginosum* slightly inhibited amylase activity at concentrations of 5 mg/mL (30.95% and 26.06% respectively) with IC_50_ values > 5 mg/mL. In other studies, polyphenol-rich extracts from different types of berries inhibited α-amylase in vitro, and the most effective were from raspberry and rowanberry (IC_50_ values of 21 and 4.5 μg/mL, respectively). Thus, strawberry and raspberry extracts were more effective amylase inhibitors than blueberry and blackcurrant [62]. Other authors observed that the extent of inhibition of α-amylase was related to appreciable amounts of soluble tannins [62]. Our fruits extracts are poor in tannins content, and it may explain the low inhibitory enzymatic activity.

#### 3.3.3. Inhibition of Pancreatic Lipase

Both fruit extracts showed no activity against lipase, while positive control Orlistat showed IC_50_ value of 0.04 μg/mL (Table 4). These results agree with a study of *P. andina* extracts that did not inhibit lipase activity [41]. The inhibition of lipase has been associated with higher content of tannins more than phenolic compounds [41]. This result could explain the absence of inhibition towards the lipase enzyme.

### 3.4. Antiproliferative Activity

Many studies reported the in vivo and in vitro effectiveness of phenolic compounds of fruits as anticancer. The bioactive compounds have significant anticancer effects through various complementary mechanisms of action, including the induction of metabolizing enzymes and the modulation of gene expression; and their impact on cell proliferation, apoptosis, and subcellular signaling pathway [63,64].

Antiproliferative activity of *E. tinifolia* and *S. lanuginosum* extracts were evaluated using three human cancer cell lines, HeLa (cervicouterine adenocarcinoma), MCF-7 (breast adenocarcinoma) and HT-29 (colon adenocarcinoma). Results showed that MCF-7 cell line was the most sensitive to the *E. tinifolia* extract, and the *S. lanuginosum* extract was against HT-29 cell line. The effect of *E. tinifolia* and *S. lanuginosum* extracts on cancer cell lines was dose-dependent and varied with the cell type and extract concentration. The concentration of 2 mg/mL of both extracts was the most effective. Nevertheless, *E. tinifolia* had the strongest effect on the three cell types. It revealed a marked decrease in the viability of the cancer cell lines after treatment. The 50% growth inhibition (IC_50_) of cells exposed to *E. tinifolia* was 0.99, 1.36 and 0.82 mg/mL, and for *S. lanuginosum* were 1.99, 3.22, and 1.97mg/mL for MCF-7, HeLa, and HT-29 cells, respectively (Table 4). It can be suggested that a wide range of phenolic compounds in the extract contributed to their cytotoxic activity. Al Hasani et al. [65] evaluated the antiproliferative activity for MCF-7 cells of *Sideroxylon mascatense* fruit extract and showed higher activity than *E. tinifolia* and *S. lanuginosum* extracts (IC_50_ value of 64 μg/mL). Though the extracts of *Pyracantha coccinea* (IC_50_ 1.2 mg/mL) and *Zosima absinthifolia* (IC_50_ 1.5 mg/mL) had less antiproliferative activity than *E. tinifolia* for HeLa cells [66,67]. The main phenol compound found on *E. tinifolia* extract was rosmarinic acid, and it could be responsible for the antiproliferative activity, as described by Niu et al. [68]. They compared the antiproliferative activity of *Rosmarinus officinalis* with rosmarinic acid and concluded that rosmarinic acid was more active than the plant extract against HeLa cells.

Breast cancer is the most frequently diagnosed cancer in women. An alternative strategy to reduce the risk of cancer is through dietary modification. *E. tinifolia* and *S. lanuginosum* extracts showed better antiproliferative activity against MCF-7 cells than an apple extract (IC_50_ = 70.7 mg/mL) [69]. Moreover, quercetin glucoside (QG) exhibited significant antiproliferative activity against MCF-7 cells. The EC_50_ value of QG in inhibiting MCF-7 cell growth was 46.4 μM [69]. This flavonoid is the most abundant phenolic compound in *S. lanuginosum* extract and could be responsible for inhibiting MCF-7 cells.

Phenolic compounds of *E. tinifolia* and *S. lanuginosum* inhibited proliferation of colon cancer cells HT-29. Similar effects have been shown of Borago extracts with CI_50_ values within the 250–300 μg/mL range after 72 h of cell exposure. Like in *E. tinifolia*, rosmarinic acid was the main phenolic compound detected in all Borago taxa [70]. In another study, the main phenolic compounds in Rabbiteye blueberry could inhibit HT-29 cancer cell proliferation and induce apoptosis. The phenolic acid fraction showed antiproliferation activities with an IC_50_ of ~1000 μg/ mL [71].

## 4. Conclusions

*Sideroxylon lanuginosum* (coma) presented the higher content of total flavonoid (21.4 ± 1.5 mg GAE/100g FW); the most abundant flavonoid detected was the quercetin glucoside. In comparison, *Ehretia tinifolia* (pinguica) had higher total phenol content (64.7 ± 2.6 mg GAE/100g FW), and the most abundant phenolic detected was rosmarinic acid. These major compounds could be related to the interesting biological activity of both extracts. *E. tinifolia* showed the best effect in antioxidant/antiradical activity both in vitro (EC_50_ = 0.32 ± 0.03 mg/mL; 4134 ± 9.7 μM TE/g dry extract), ex vivo assay (IC_50_ = 58.55 ± 2.4 μg/mL), and the inhibition of α-glucosidase (IC_50_ = 0.17 ± 0.1 mg/mL). Additionally, the extract of *E. tinifolia* showed a better effect on the growth inhibition of cancer cells, particularly against those of the human colon (IC_50_ = 0.82 ± 0.09 mg/mL) and breast cancer (IC_50_ = 0.99 ± 0.01 mg/mL). These results contribute to the scant information available on these wild fruits and contribute to revalue their use as a natural source of antioxidants with potential therapeutic application in diseases caused by free radicals. Further investigations using animal models are needed to confirm the beneficial effects of the studied extracts as supplementary treatments for oxidative diseases.

## Figures and Tables

**Table 1:** Total phenolic content, total flavonoid content, and antioxidant/antiradical capacities of *Ehretia tinifolia* and *Sideroxylon laniginosum* extracts.

Sample	TPC mg GAE/100g F.W.	TFC mg CE/100g F.W.	TEAC μmol TE/g	DPPH EC_50_ mg/mL	Hemolytic Inhibition IC_50_ (μg/mL)
*S. lanuginosum*	21.4 ± 1.5 ^b^	6.42 ± 0.9 ^a^	4134 ± 97 ^a^	0.48 ± 0.05 ^a^	61.76 ± 7.9 ^b^
*E. tinifolia*	64.7 ± 2.6 ^a^	5.1 ± 0.4 ^b^	2454 ± 38 ^b^	0.32 ± 0.03 ^b^	58.55 ± 6.5 ^b^
** Control	—	—	—	0.013 ± 2 ^c^	289 ± 20 ^a^

** Trolox was used on DPPH free radical scavenging assay and ascorbic acid on AAPH-induced hemolysis assay. *n* = 3, (a–c) on each column show significant differences among each determination, according to Tukey’s test (*p* < 0.05).

**Table 2:** Tentative identification of phenolic compounds of *E. tinifolia* fruit extract, determined by HPLC-DAD-MS/MS.

Peak	Rt (min)	UV Max	[M+H]^+^	MS/MS Fragments	Tentative Identification
1	5.46	261	297.4	135	Unknown
2	7.01	281	191.2	173, 129, 111	Quinic acid
3	7.53	262	373.0	311, 285, 267, 249, 241, 227, 196	Unknown
4	7.98	252, 275 (sh)	282.5	150, 133	Unknown
5	8.07	301	331.3	169, 125	Galloil-glucose
6	8.58	282	507.2	489, 459, 293, 233, 195, 131, 125,113	Caffeic acid derivative
7	8.74	280, 314 (sh)	165.0	137	Unknown
8	8.96	279	507.2	233, 165, 150, 125	Unknown
9	9.08	276	719.0	515, 359, 197, 179, 135	Rosmarinic acid derivative
10	9.38	272	515.0	269, 251, 225, 213, 179, 159, 135, 109	Unknown
11	9.60	278	359.0	197, 179, 135	Syringic acid hexoside
12	10.00	281	521.0	197, 179, 135	Syringic acid dihexoside
13	11.03	272	165.0	150, 121	Unknown
14	13.28	278, 320 (sh)	401.0	359, 341, 297, 197, 179,135	Unknown
15	14.78	278, 314	365.4	323, 262, 250	Unknown
16	15.06	311, 292 (sh)	373.0	211, 179, 123	Methyl rosmarinate
17	15.35	281, 330 (sh)	567.0	179, 135	Caffeic acid derivative
18	19.45	291, 322	179.0	135	Caffeic acid
19	25.04	276	863.0	701, 521, 359, 315, 297, 197, 135	Syringil-rosmarinic acid dihexoside
20	28.48	276	695.5	579, 554, 537, 493, 312, 295, 277, 203, 135	Lithospermic acid derivative
21	30.38	276	695.5	579, 554, 537, 493, 312, 295, 277, 203, 135	Lithospermic acid derivative
22	31.69	276	695.5	579, 554, 537, 493, 312, 295, 277, 203, 135	Lithospermic acid derivative
23	32.35	276	695.5	579, 554, 537, 493, 312, 295, 277, 203, 135	Lithospermic acid derivative
24	34.22	276	695.5	579, 554, 537, 493, 312, 295, 277, 203, 135	Lithospermic acid derivative
25	35.01	276	695.5	579, 554, 537, 493, 312, 295, 277, 203, 135	Lithospermic acid derivative
26	45.25	275, 330 (sh)	597.4	579, 509, 491, 355, 337, 329, 311, 293, 267, 239, 197, 179, 135, 109	Unknown
27	46.49	284, 320	861.5	843, 817, 655, 521, 501, 475, 457, 383, 359, 339, 323, 197, 179, 135	Rosmarinic acid derivative
28	47.31	285, 318	521.0	359, 197, 179, 161, 135	Rosmarinic acid-hexoside
29	47.94	283, 327	521.0	359, 197, 179, 161, 135	Rosmarinic acid-hexoside
30	51.02	330, 290 (sh)	359.0	197, 179, 161, 135	Rosmarinic acid
31	53.13	278	537.0	493, 359, 295, 277, 203, 185, 159, 135, 109	Lithospermic acid A

sh = shoulder.

**Table 3:** Tentative identification of phenolic compounds of *S. lanoginosum* fruit extract by HPLC-DAD electrospray ionization (ESI)-MS/MS.

Peak	Rt (Min)	UV Max	[M+H]^+^	MS/MS Fragments	Tentative Identification
1	8.44	272	169.1	125, 113	Gallic acid
2	8.76	245	137.0		*p*-hydroxybenzoic acid
3	8.89	278	329.3	167, 151, 109	Unknown
4	9.30	282	331.2	169, 125	Unknown
5	9.74	283	315.2	152, 108	Unknown
6	11.02	259-293	153.1	123, 109	Protocatechuic acid
7	11.43	286, 315 (sh)	461.0	351, 323, 248, 233, 193	Ferulic acid derivative
8	12.11	285, 324 (sh)	463.3	283, 272, 255, 175, 163	Unknown
9	13.48	295, 311	487.4	187, 163, 145, 119	Coumaric dihexoside
10	14.15	256, 311 (sh)	435.2	241, 193, 153	Ferulic acid derivative
11	15.26	255, 335	311.3	249, 231, 205, 187, 161, 14 147, 135, 121	Unknown
12	15.82	303	421.2	241	Unknown
13	16.41	280	417.1	399, 227, 167, 153	Unknown
14	17.77	281, 310 (sh)	387.0	163	Coumaric acid derivative
15	22.88	267, 327 (sh)	241.2	197, 168, 141, 130	Phenolic acid derivative
16	25.63	262, 331 (sh)	295.0	251, 189, 137, 121	Phenolic acid derivative
17	29.23	307, 290 (sh)	163.0	119	Coumaric acid
18	32.95	255, 351, 301 (sh)	755.5	609, 489, 355, 343, 325 301, 271, 179	Quercetin glucoside dirhamnoside
19	34.82	354, 300 (sh)	479.0	317, 287, 271, 179, 151	Myricetin glucoside
20	35.54	254, 352, 302 (sh)	771.0	301	Quercetin diglucoside rhamnoside
21	43.66	256, 353, 305 (sh)	609.4	343, 301, 271, 255, 179, 151	Quercetin neohesperidoside
22	44.73	256, 355, 301 (sh)	463.4	306, 301, 271, 255, 248 179, 151, 121	Quercetin glucoside
23	45.40	257, 342, 302 (sh)	477.0	301, 151	Quercetin glucuronide
24	45.68	256, 354, 300 (sh)	477.0	301, 179, 151	Quercetin glucuronide
25	46.68	267, 359 (sh)	433.8	301, 271, 179, 151	Quercetin pentoside
26	47.52	255, 351	433.9	301, 271, 256, 180, 152	Quercetin pentoside
27	47.71	264, 349	447.9	285, 256, 227, 151	Kaempferol glucoside
28	47.99	284, 340 (sh)	436.0	346, 316, 274, 167, 123	Unknown
29	49.06	257, 347	447.0	301, 273, 257, 179, 151	Kaempferol glucoside
30	50.85	371	317.0	179, 151, 138	Myricetin
31	51.18	327, 287 (sh)	359.0	197, 179, 161, 135	Rosmarinic acid
32	54.79	264, 316, 356 (sh)	609.8	463, 301, 151	Quercetin rutinoside
33	55.25	251, 330, 300 (sh)	639.8	477, 463, 316, 300	β-hydroverbascoside
34	57.00	370, 300 (sh)	301.2	273, 229, 179, 161, 151, 121	Quercetin

sh = shoulder.

**Table 4:** Inhibition of digestive enzymatic and antiproliferative activity by *E. tinifolia* and *S. lanuginosum* fruit extracts.

Sample	Half Maximal Inhibitory Concentration [mg/mL]						
	α-Glu	α-Amy	Lipase	MCF-7	HeLa	HT-29	* RBCs
*S. lanuginosum*	0.21 ± 0.3 ^a^	>5	>5	1.99 ± 0.3 ^a^	3.22 ± 0.8 ^a^	1.97 ± 0.2 ^a^	>5
*E. tinifolia*	0.17 ± 0.1 ^a,b^	>5	>5	0.99 ± 0.01^b^	1.36 ± 0.2 ^b^	0.82 ± 0.09 ^b^	>5
** Control	0.13 ± 0.2 ^b^	0.97 ± 0.08	0.17 ± 0.20	0.013 ± 0.001 ^c^	0.011 ± 0.002 ^c^	0.015 ± 0.001 ^c^	ND

* RBCs = Red blood cells = Erythrocytes. ** Acarbose was used on α-glucosidase and α-amylase assays, orlistat on Lipase inhibition and taxol against cancer cells lines. *n* = 3, literals (a–c) on each column show significant differences among treatments, according to Tukey’s test (*p* < 0.05). ND: not determined.
